# Limited recurrence distance of glioblastoma under modern radiotherapy era

**DOI:** 10.1186/s12885-021-08467-3

**Published:** 2021-06-22

**Authors:** Ziwei Tu, Huifen Xiong, Yang Qiu, Guoqing Li, Li Wang, Shiyi Peng

**Affiliations:** 1 NHC Key Laboratory of Personalized Diagnosis and Treatment of Nasopharyngeal Carcinoma (Jiangxi Cancer Hospital of Nanchang University), 519 East Beijing Road, Nanchang, 330029 China; 2 Department of Radiotherapy, Eye & ENT Hospital, Shanghai Medical College, Fudan University, Shanghai, China

**Keywords:** Glioblastoma multiforme (GBM), Radiotherapy, Recurrent distance, Peritumoral edema

## Abstract

**Background:**

The optimal treatment volume for Glioblastoma multiforme (GBM) is still a subject of debate worldwide. The current study was aimed to determine the distances between recurring tumors and the edge of primary lesions, and thereby provide evidence for accurate target area delineation.

**Methods:**

Between October 2007 and March 2019, 68 recurrent patients with GBM were included in our study. We measured the distance from the initial tumor to the recurrent lesion of GBM patients by expanding the initial gross tumor volume (GTV) to overlap the center of recurrent lesion, with the help of the Pinnacle Treatment Planning System.

**Results:**

Recurrences were local in 47(69.1%) patients, distant in 12(17.7%) patients, and both in 9(13.2%) patients. Factors significantly influencing local recurrence were age (*P* = 0.049), sex (*P* = 0.049), and the size of peritumoral edema (*P* = 0.00). A total number of 91 recurrent tumors were analyzed. All local recurrences occurred within 2 cm and 94.8% (55/58) occurred within 1 cm of the original GTV based on T1 enhanced imaging. All local recurrences occurred within 1.5 cm and 98.3%(57/58) occurred within 0.5 cm of the original GTV based on T2-FLAIR imaging. 90.9% (30/33) and 81.8% (27/33) distant recurrences occurred >3 cm of T1 enhanced and T2-Flair primary tumor margins, respectively.

**Conclusions:**

The 1 cm margin from T1 enhanced lesions and 0.5 cm margin from T2-Flair abnormal lesions could cover 94.8 and 98.3% local recurrences respectively, which deserves further prospective study as a limited but effective target area.

**Supplementary Information:**

The online version contains supplementary material available at 10.1186/s12885-021-08467-3.

## Introduction

Glioblastoma multiforme (GBM) is a common type malignancy with dismal prognosis in the primary brain tumors worldwide [1–3]. The current standard therapy regiments include maximal gross tumor resection followed by radiotherapy (RT) plus concurrent and adjuvant temozolomide [4–7]. Numerous randomised controlled trials have demonstrated the significant benefits of radiotherapy in initial treatment of glioblastoma patients, which positions it as the cornerstone of adjuvant treatment for decades [8, 9].

The target region of radiation therapy have developed from the whole brain to partial-brain, given the pace of progress on these and other researches including the Brain Tumor Cooperative Group 80–01 randomized trial [10]. However, some disputes exist in the optimal treatment volume for GBM among medical institutions. For example, the European Organization for Research and Treatment of Cancer (EORTC) employs a single target volume with 2–3 cm radiation margins around the primary tumor bed (based on abnormalities on post-contrast MRI) [4]. Nevertheless, the Radiation Therapy Oncology Group (RTOG) implements the initial clinical target volume (CTV) and the boost field separately by adding a 2-cm margin to postoperative peritumoral edema and residual tumor [11]. The University of Texas MD Anderson Cancer Center adds a 2 cm margin around the gross tumor volume (GTV) to generate CTV, which comprises the surgical cavity and the postoperative residual tumor, while excluding any edema. A 5-mm margin was added to the CTV and GTV to create the PTV and boost PTV, respectively [12]. Adult Brain Tumor Consortium guidelines (ABTC) defines the initial planning target volume as an expansion of 1 cm from T1-enhancing tumor plus peritumoral edema, and the boost planning target volume as an expansion of 1 cm just from T1-enhancing tumor [13, 14].

Interestingly, these different treatment standards mentioned above have similar risk of marginal recurrences. Even when a PTV boost margin of 1 cm or less was used, the recurrence pattern of patients with glioblastoma stays much the same [14–17]. Limited margin size could reduce the total volume of normal brain irradiated compared to the radiation therapy plans including peritumoral edema. Therefore, we desperately need further evidence to formulate a more limited but effective radiation area, which could potentially reduce acute and late neurotoxicity with a decrease in marginal or distant recurrences.

In this study, we devoted to examining the distance between the recurrent lesion and initial tumor of GBM patients that have been treated during an era of local conformal radiotherapy, which will provide a basis for the contouring of the target volume.

## Materials and methods

### Selection of patients

The records of 68 recurrent patients treated for GBM at Jiangxi Cancer Hospital between October 2007 and March 2019 were retrospectively assessed. Detailed imaging data before and after treatment, radiation dosimetry records, and the outcome of treatment were available for all patients in our study. Clinical information were collected including age, sex, KPS, tumor patterns, tumor volume, SVZ involvement, peritumoral edema, extension of resection, time from surgery to radiotherapy, and use of chemotherapy (Table 1).

**Table 1:** Characteristics of 68 patients with glioblastoma

Characteristics	Patient number	Local recurrence	Distant recurrence	χ2	P
Sex					
Male	41	32	9	3.859	0.049^*^
Female	27	15	12		
Age					
≤ 45y	27	15	12	3.859	0.049^*^
>45y	41	32	9		
KPS					
≥ 90	39	26	13	0.257	0.612
<90	29	21	8		
Tumor					
Unifocal	59	40	19	0.047	0.829
Multifocal	9	7	2		
Extension of resection					
Subtotal	33	24	9	0.391	0.532
Total	35	23	12		
Chemotherapy					
No	10	7	3	0	1
Yes	58	40	18		
SVZ involvement					
Yes	45	30	15	0.374	0.541
No	23	17	6		
Peritumoral edema					
>1.8 cm	41	35	6	12.771	0.00^*^
≤ 1.8 cm	27	12	15		
Tumor volume					
>41cm^3^	34	27	7	3.376	0.066
≤ 41 cm^3^	34	20	14		
Time from surgery to radiotherapy					
<40D	48	35	13	1.103	0.294
≥ 40D	20	12	8		

### Treatment and follow-up

The patients were irradiated according to The University of Texas MD Anderson Cancer Center guidelines. Radiotherapy plans were normalized so 95% of the isodose line completely covers PTV. Patients underwent MRI scans at the end of radiotherapy and then followed every 2–3 months. The imaging sequences included at least pre- and post-contrast T1, T2, and FLAIR. The MR diffusion weighted imaging, magnetic resonance spectroscopy and perfusion imaging were conducted in part of patients.

### Recurrences analysis

Postoperative and recurrent MR images were co-registered to the treatment planning CT in Pinnacle Treatment Planning System. The recurrent patterns were determined by the volume of recurrent T1-enhancing tumor present within the 95% isodose line of the prior boost plan. The recurrent lesions were defined as “in-field” if > 80% of the intersecting area was covered by the 95% isodose line, “marginal” if 20–80% of the intersecting region was inside of the 95% isodose line, or “distant” if< 20% of the intersectant volume was included in the 95% isodose line [13]. In field and marginal lesions were defined as local recurrences. We calculated the center of each recurrent tumor in the Pinnacle Treatment Planning System, and then expanded the primary tumor according to the edge of initial T1 contrast-enhancing and T2-Flair lesion. The distance when the curve of the expansion passed right through the center was defined as DT1 and DT2 (as described in Fig. 2).

### Statistics

The threshold value of patient characteristics such as tumor volume and peritumoral edema etc. is obtained by ROC curve. Chi-square analysis was used to investigate differences in clinical features of local and distant recurrent patients. The Kaplan-Meier method was applied to estimate the overall survival (OS) and progression-free survival (PFS). The results of categorical variables were analysed by SPSS, version 22.

## Results

Sixty-eight cases of recurrent GBM initially treated with postoperative radiotherapy between October 2007 and March 2019 were available for analysis. The baseline and treatment characteristics were collected in Table 1. The median age was 44 years old (range, 17–71 years). 51.5%(35/68)patients underwent a gross total resection with 85%(58/68) postoperative chemotherapy. Notably, local recurrence was significantly associated with older age (*P* = 0.049), fewer females (*P* = 0.049), and peritumoral edema>1.8 cm (*P* = 0.00).

**Fig. 1:**
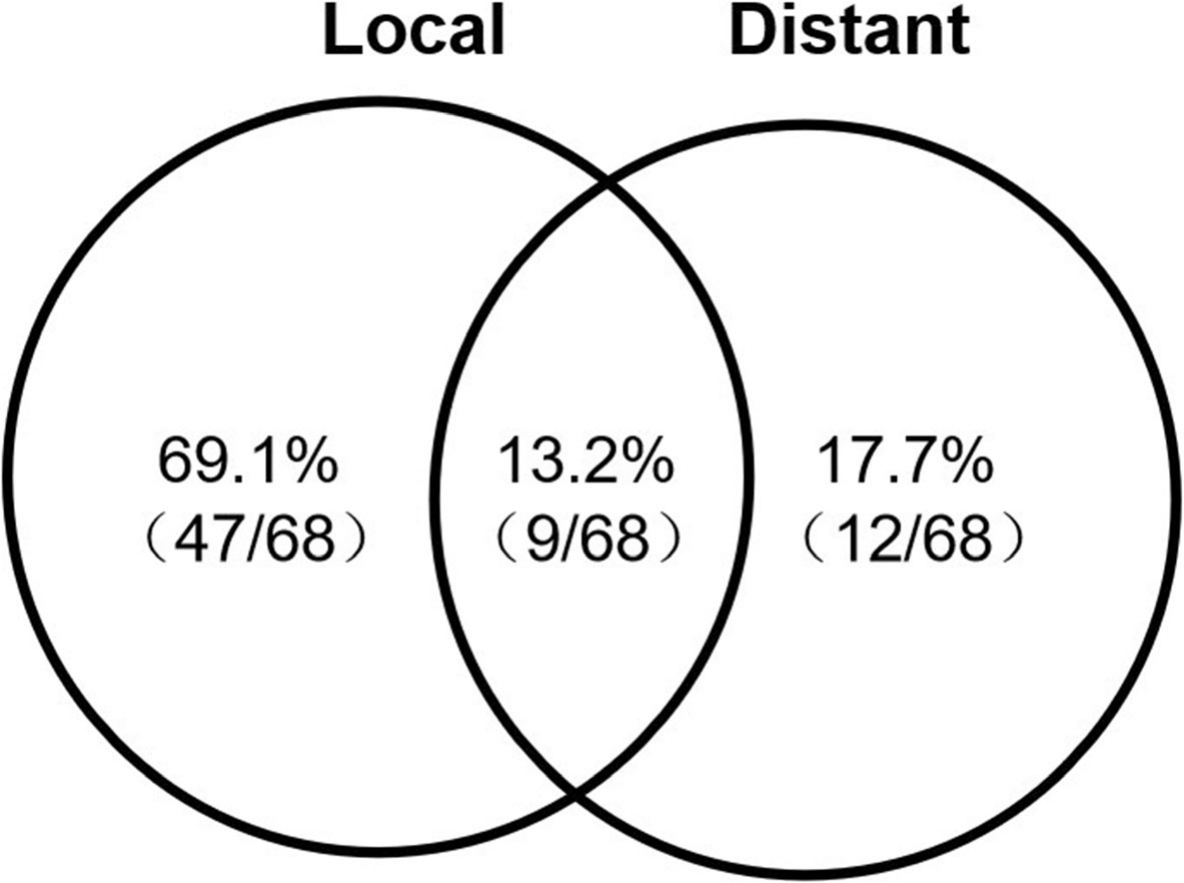
The pattern of failure. 69.1% (47/68) had local recurrence, 17.7% (12/68) had distant recurrence, and 13.2% (9/68) had both local and distant recurrence simultaneously

The median overall survival (OS) was 13 months (range 3 to 92 months). The 24-month survival rates were 21%. The median progression-free survival (PFS) was 7 months (range 1 to 78 months). The 24-month PFS rates were 13% (Supplementary 1). Figure 1 showed **t**he pattern of failure related to the treated volumes. In the 68 recurrent cases, 47(69.1%) had local recurrence, 12(17.7%) had distant recurrence, and 9(13.2%) had both local and distant recurrence simultaneously.

**Fig. 2:**
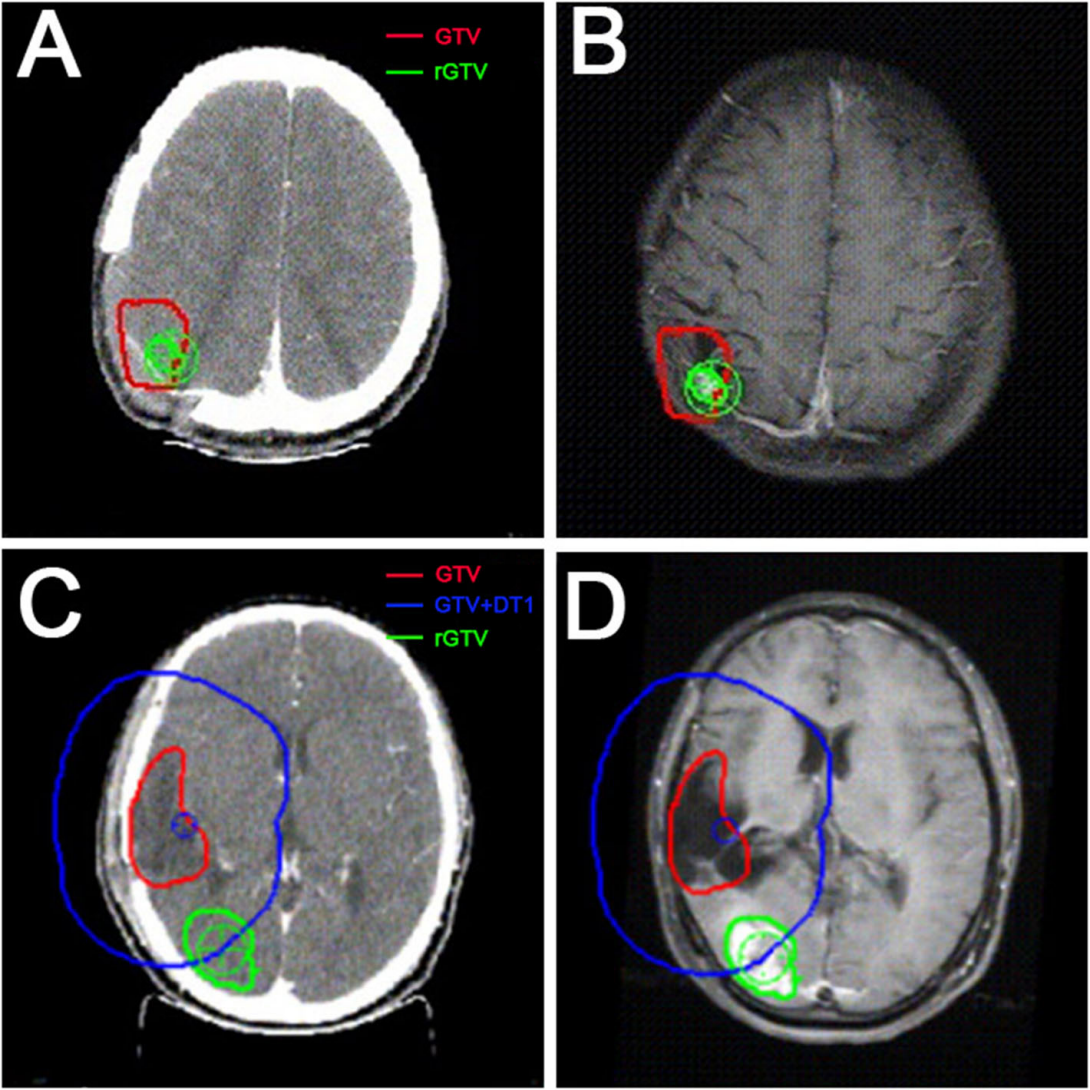
**A, B** case 1: example of local failure. The GTV (red line) showed the margin of primary tumor. The rGTV (green line) showed the local recurrent lesion, and the center of rGTV (green cross) is inside the GTV. **C, D** case 2: example of distant failure. The GTV (red line) showed the margin of primary tumor and the rGTV (green line) showed the distant recurrent lesion. The GTV + DT1 (blue line) was delineated to measure the distance between the GTV and the center of rGTV (green cross). In this patient, the distance was 2.9 cm

**Fig. 3:**
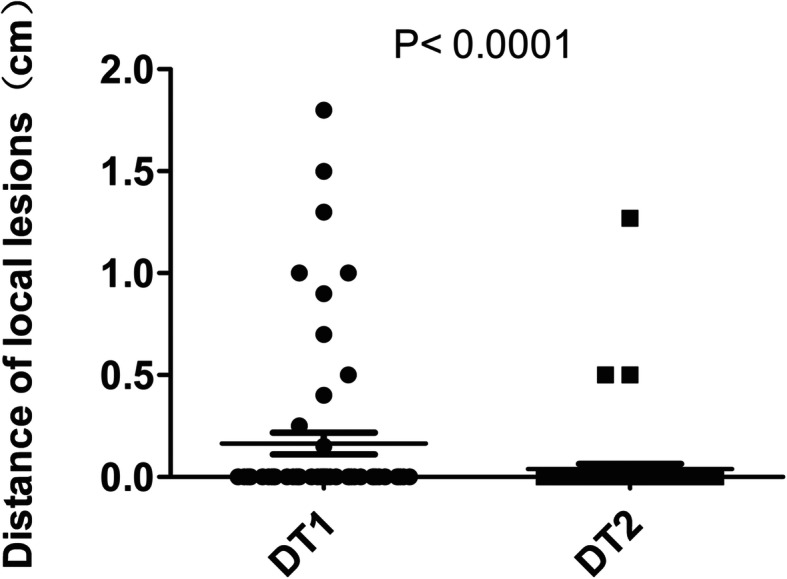
The distance from the edge of primary tumor on T1 enhanced signal (DT1) and the abnormal T2 signal (DT2) MRI scan to the corresponding center of the 58 local lesions

**Fig. 4:**
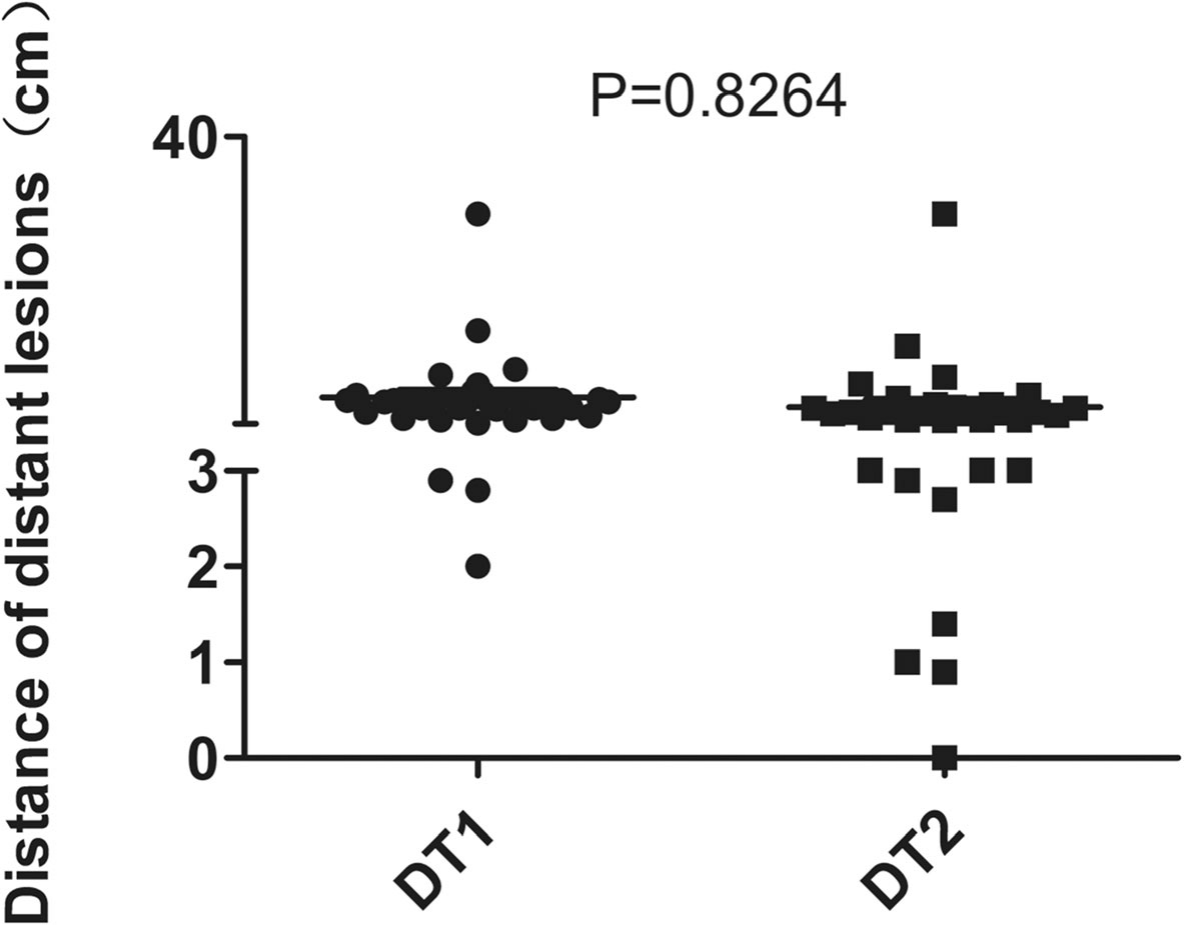
The distance from the edge of primary tumor on T1 enhanced signal (DT1) and the abnormal T2 signal (DT2) MRI scan to the corresponding center of the 33 distant lesions

The total number of recurrent lesions of the 68 patients was 91 with 58 local and 33 distant lesions. We measured and recorded the distance separately from T1 contrast-enhancing (DT1) and T2-Flair (DT2) original tumor margin to the center of recurrent lesions. A local and a distant recurrent example patient cases are displayed in Fig. 2. As shown in Fig. 3 and Table 2, the percentage of DT1 within 2 cm, 1.5 cm and 1 cm was 100% (58/58), 98.3% (57/58) and 94.8% (55/58) respectively. All of the DT2 was within 1.5 cm and 98.3% (57/58) of the DT2 was within 0.5 cm. Among the 33 distant lesions (Fig. 4 and Table 3), 90.9% (30/33) of the DT1 and 81.8% (27/33) of the DT2 were ≥ 3 cm, respectively. The only 2-cm DT1 distant lesion was located in the contralateral brain parenchyma.

**Table 2:** Distance from the center of local recurrences to the edge of primary lesions

	Local lesions(%)
Distance to the edge of the TI enhanced signal (DT1)	
DT1 ≤ 1 cm	55 (94.8%)
1 < DT1 ≤ 1.5 cm	2 (3.5%)
1.5 < DT1 ≤ 2 cm	1 (1.7%)
Distance to the edge of the abnormal T2 signal (DT2)	
DT2 ≤ 1 cm	57 (98.3%)
1 < DT2 ≤ 1.5 cm	1 (1.7%)
1.5 < DT2 ≤ 2 cm	0 (0%)

**Table 3:** Distance from the center of distant recurrences to the edge of primary lesions

	Distant lesions(%)
Distance to the edge of the TI enhanced signal (DT1)	
DT1 ≤ 2 cm	1 (3%)
2 < DT1 < 3 cm	2 (6.1%)
DT1 ≥ 3 cm	30 (90.9%)
Distance to the edge of the abnormal T2 signal (DT2)	
DT2 ≤ 2 cm	4 (12.1%)
2 < DT2 < 3 cm	2 (6.1%)
DT2 ≥ 3 cm	27 (81.8%)

## Discussion

Although RT remains an important and essential therapy for GBM patients, no uniform target delineation guideline has been established worldwide, leading to immense variation of the treatment volume for GBM patients in different cancer centers. According to Gebhardt et al., for a round tumor with a 5 cm radius, an expansion of 1.0 cm from GTV to PTV will generate a total irradiation volume of 452 cm^3^. Furthermore, a 2.5 or 3.5 cm total margin will lead to an increase in the consequent treatment volume to 707 or 908 cm^3^, respectively. Obviously, adding the margin to 2.5 cm would increase the target volume by more than twice, which may result in additional toxicity to the patients [13]. Therefore, we urgently need an accurate expansion distance from GTV to CTV.

Early pattern-of-failure studies showed that 70–90% recurrent lesions occurred within 2–3 cm of the primary tumor, even after whole-brain radiotherapy [18–24]. However, as revealed by McDonald, several limitations inevitably exist in these early studies. Firstly, the data of these studies is based on early-generation CT technology, which has inferior image resolution compared with MRI. Secondly, the assessment of recurrences required cumbersome manual detection between the radiation plan and subsequent imaging and only one or a few slices were usually selected for measuring two-dimensionally, instead of stereoscopically. Moreover, Comparisons were performed on preoperative CT images, whereas contemporary treatment planning was sketched from the postoperative imaging [15] . Owing to the use of planning system, we accurately calculated the center of the recurring tumor, and obtained the distance of the three-dimensional expansion from the edge of the primary tumor to the center, which made up for these deficiencies.

Our results showed that 90.9% (30/33) of distant recurrences occurred outside 3 cm of the original T1 enhanced lesions, 87.9% (29/33) occurred outside 2 cm of the original T2-FLAIR abnormal lesions, which exceeded the current target area of most treatment centers. By contrast, all local recurrences occurred within 2 cm and 94.8% occurred within 1 cm of the original T1 enhanced lesions, all but 1(1.7%) local recurrences occurred within 0.5 cm of the original T2-FLAIR abnormal lesions. It seems that the current diverse target area delineation guidelines can mainly cover local failures, for which the 1 cm margin from T1 enhanced lesions and the 0.5 cm margin fromT2-Flair abnormal lesions are expected to be enough. Actually, over the years, several cancer centers have published their treatment experiences on limited-margin radiotherapy. Gebhardt et al. studied the patterns of failure in 95 GBM patients treated with limited-margin according to Adult Brain Tumor Consortium guidelines. The boost and initial target volume included a 1 cm expansion based on the T1-enhancing or T2-flair imaging. Among the 95 recurrent patients, 77 (81%) suffered an in-field relapse, 6 (6%) had a marginal recurrence, and 27 (28%) experienced a distant dissemination. Low incidence of marginal recurrence indicated limited margins may have a negligible effect on the recurrence pattern [13]. Consistantly, Wake Forest examined 161 patients irradiated with 5-, 10, and 15- 20 mm CTV margins, and concluded no statistic difference in patterns of recurrence, PFS or OS among patients with various expansion distances [25].

Deserved to be mentioned, our study revealed that obvious edema (>1.8 cm) before radiotherapy indicates more local recurrence(85.3% vs 44.4%, *P* = 0.00). However, the impact of peritumoral edema on the survival of glioma patients is controversial. The effect of peritumoral edema on the prognosis and recurrence pattern of patients with glioblastoma remains inconclusive. No significant difference in outcome was found in two prospective international studies that allowed application of EORTC and RTOG guidelines [26, 27]. In other words, inclusion of the whole FLAIR hyperintense region or not, did not confer a prognostic benefit. Chang et al. conducted a series of studies on whether the peritumoral edema should be included in the CTV delineation of glioblastoma [12], which revealed identical recurrence pattern between the two sets of radiation plans. However, we believe that these studies cannot completely rule out the effect of edema on the recurrence patterns of glioblastoma, because even if CTV only comes from the expansion of the MRI enhanced lesion, the edema region was still, more or less included. Other factors significantly influencing local recurrence include age (*P* = 0.049) and sex (*P* = 0.049), which need to be confirmed by more studies. Besides, the tumor volume data is very close to significance (*P* = 0.066) and might become significant with increased sample size.

However, there are several limitations in our study. The small sample size restricted the number of variables analyses. In addition, some patients didn’t complete chemotherapy due to the poor economic conditions. Other limitations of the present study stem from the retrospective design. We need to prospectively compare the different planning methods in terms of efficacy and risk of late radiation-induced toxicity.

## Conclusion

Most of distant recurrences occurred outside 3 cm of the original lesions, which is beyond the current treatment volume of most cancer centers. CTV based on 2 cm margin of original tumor on MRI scan could well cover the site of local recurrence in all cases. The 1 cm margin from T1 enhanced lesions and 0.5 cm margin from T2-Flair abnormal lesions could cover 94.8 and 98.3% local recurrences respectively, which deserves further prospective study as a limited but effective target area.

## Supplementary Information

**Additional file 1 Supplementary 1** Kaplan–Meier analysis of overall survival (OS) and progression-free survival (PFS).

**Additional file 2.** Univariate and Multivariate analysis of factors associated with OS. Univariate and Multivariate analysis of factors associated with PFS.

**Additional file 3.**

## Data Availability

We could share the detailed data. You could contact Ziwei Tu (email:tuziwei198803@163.com), if someone wants to request the data from this study.

## Abbreviations

GBM: Glioblastoma multiforme
GTV: Gross tumor volume
RT: Radiotherapy
EORTC: European organization for research and treatment of cancer
RTOG: Radiation therapy oncology group
CTV: Clinical target volume
ABTC: Adult brain tumor consortium guidelines
OS: Overall survival
PFS: Progression-free survival
DT1: The distance separately from T1 contrast-enhancing
DT2: The distance separately from T2-Flair
SVZ: Subventricular zone
