# Supplementary material for: Limited recurrence distance of glioblastoma under modern radiotherapy era
Source: BMC Cancer. 2021 Jun 22;21:720. doi: 10.1186/s12885-021-08467-3 (PMC8218451; doi:10.1186/s12885-021-08467-3)
Supplement: Supplementary file 1 — Additional file 1 Supplementary 1 Kaplan–Meier analysis of overall survival (OS) and progression-free survival (PFS). [file 12885_2021_8467_MOESM1_ESM.docx]

**Supplementary 1** Kaplan–Meier analysis of overall survival (OS) and progression-free survival (PFS).
