# Supplementary material for: Limited recurrence distance of glioblastoma under modern radiotherapy era
Source: BMC Cancer. 2021 Jun 22;21:720. doi: 10.1186/s12885-021-08467-3 (PMC8218451; doi:10.1186/s12885-021-08467-3)
Supplement: Supplementary file 2 — Additional file 2. Univariate and Multivariate analysis of factors associated with OS. Univariate and Multivariate analysis of factors associated with PFS. [file 12885_2021_8467_MOESM2_ESM.docx]

**Univariate and Multivariate analysis of factors associated with OS**

| Variables |  | Univariate analysis | | |  | Multivariate analysis | | |
| --- | --- | --- | --- | --- | --- | --- | --- | --- |
|  | HR | | *95%CI* | *P^a^* |  | HR | *95%CI* | *P^a^* |
| Gender  Male *vs* Female | 1.134 | | 0.650-1.979 | 0.657 |  | 1.229 | 0.666-2.270 | 0.509 |
| Age(years)  ≤45y vs＞45y | 0.906 | | 0.687-1.197 | 0.488 |  | 0.814 | 0.596-1.114 | 0.199 |
| KPS  ≥90 vs ＜90 | 0.770 | | 0.583-1.018 | 0.067 |  | **0.700** | **0.519-0.945** | **0.020** |
| Extension of resection  Subtotal vs Total | 0.845 | | 0.644-1.110 | 0.227 |  | 0.735 | 0.515-1.047 | 0.088 |
| Chemotherapy  Yes vs No | 1.075 | | 0.759-1.522 | 0.683 |  | 1.110 | 0.749-1.644 | 0.602 |
| Peritumoral edema  ＞1.8cm vs ≤1.8cm | 0.930 | | 0.704-1.229 | 0.612 |  | 1.030 | 0.700-1.515 | 0.881 |
| Tumor volume  ＞41cm³ vs ≤41cm³ | 1.149 | | 0.876-1.507 | 0.316 |  | **1.413** | **1.015-1.966** | **0.041** |
| Time from surgery  to radiotherapy  ＜40D vs ≥40D | 0.922 | | 0.690-1.233 | 0.586 |  | 0.827 | 0.598-1.143 | 0.249 |
| Recurrence pattern  Local vs Distant | 0.825 | | 0.623-1.091 | 0.177 |  | **0.618** | **0.424-0.900** | **0.012** |

Abbreviation: OS, overall survival; 95% CI, 95% conﬁdence interval; HR, hazard ratio.

1. Cox proportional hazards model;

**Univariate and Multivariate analysis of factors associated with PFS**

| Variables |  | Univariate analysis | | |  | Multivariate analysis | | |
| --- | --- | --- | --- | --- | --- | --- | --- | --- |
|  | HR | | *95%CI* | *P^a^* |  | HR | *95%CI* | *P^a^* |
| Gender  Male *vs* Female | 1.014 | | 0.616-1.671 | 0.956 |  | 0.835 | 0.487-1.431 | 0.511 |
| Age(years)  ≤45y vs＞45y | 0.994 | | 0.777-1.271 | 0.963 |  | 1.047 | 0.805-1.362 | 0.733 |
| KPS  ≥90 vs ＜90 | 0.871 | | 0.682-1.114 | 0.271 |  | 0.863 | 0.669-1.114 | 0.259 |
| Extension of resection  Subtotal vs Total | **0.686** | | **0.531-0.887** | **0.004** |  | **0.729** | **0.543-0.978** | **0.035** |
| Chemotherapy  Yes vs No | 1.222 | | 0.869-1.719 | 0.250 |  | 1.175 | 0.817-1.689 | 0.384 |
| Peritumoral edema  ＞1.8cm vs ≤1.8cm | **0.742** | | **0.570-0.964** | **0.026** |  | 0.884 | 0.637-1.226 | 0.460 |
| Tumor volume  ＞41cm³ vs ≤41cm³ | 1.172 | | 0.918-1.497 | 0.203 |  | 1.053 | 0.795-1.395 | 0.720 |
| Time from surgery  to radiotherapy  ＜40D vs ≥40D | 1.054 | | 0.808-1.373 | 0.699 |  | 1.002 | 0.760-1.320 | 0.989 |
| Recurrence pattern  Local vs Distant | 1.186 | | 0.910-1.546 | 0.207 |  | 1.090 | 0.801-1.484 | 0.584 |

Abbreviation: PFS, Progression free survival; 95% CI, 95% conﬁdence interval; HR, hazard ratio.

1. Cox proportional hazards model;
